# Parental acceptance of behaviour guidance techniques used with Thai autistic patients in dental practice

**DOI:** 10.1007/s40368-025-01123-5

**Published:** 2025-10-21

**Authors:** A. Manopetchkasem, P. Leelataweewud, N. Srimaneekarn, A. Smutkeeree

**Affiliations:** Faculty of Dentistry, Mahidol University, Bangkok, Thailand

**Keywords:** Parental acceptance, Autistic, Behaviour guidance techniques, Dental treatment

## Abstract

**Purpose:**

To evaluate parental acceptance of nine behaviour guidance techniques (BGTs) for Thai autistic patients in dental treatment and to assess the factors associated with this acceptance.

**Methods:**

A cross-sectional study was conducted among 110 parents of autistic patients using an online questionnaire. The study examined nine BGTs: tell–show–do (TSD), positive reinforcement (PR), distraction (DIS), nitrous oxide/oxygen inhalation (NOOI), active restraint by parent (ARBP), active restraint by staff (ARBS), passive restraint by device (PRBD), oral sedation (OS), and general anaesthesia (GA). The questionnaire collected demographic data and measured parental acceptance using a visual analog scale (VAS) from 0 to 100 after participants viewed portraying each BGT in scenarios simulating potentially cooperative autistic patients. Data were analysed using the Mann–Whitney *U* test, Kruskal–Wallis test with Bonferroni correction and linear regression analysis.

**Results:**

All BGTs received mean VAS score above 60. PR was rated highest, followed by DIS, TSD, PRBD, ARBS, ARBP, GA, OS, and NOOI. Parental acceptance was significantly influenced by previous BGTs experience, past dental experiences, autistic severity level, and parental education level.

**Conclusion:**

All BGTs evaluated were generally accepted by parents of autistic patients in Thailand. PR was the most accepted technique, whereas NOOI received the lowest acceptance.

## Introduction

Autism is a neurodevelopmental condition characterized by deficits in social interaction, communication, and repetitive behaviours or restricted interests (Manning-Courtney et al. 2003; APA 2013; Chandrashekhar and Bommangoudar 2018). Patients with autism are more likely to experience periodontal problems, often resulting from poor oral hygiene and challenges in maintaining oral cleanliness (Shapira et al. 1989; Bartolomé-Villar et al. 2016; da Silva et al. 2017). However, the rate of dental caries occurrence in this population remains controversial; some studies report a lower prevalence, while others report a higher rate as compared with healthy subjects (Shapira et al. 1989; Bartolomé-Villar et al. 2016; da Silva et al. 2017).

Individuals with autism display distinct behavioural patterns and require different behaviour guidance techniques (BGTs) compared to nonautistic individuals (Shapira et al. 1989; da Silva et al. 2017; Chandrashekhar and Bommangoudar 2018; Taylor et al. 2020; Yaguchi and Hidaka 2020). Barriers to receiving dental treatment for patients with autism include difficulties in managing emotions, repetitive body movements, hyperactivity associated with attention deficits, and a low frustration threshold (Chandrashekhar and Bommangoudar 2018). The American Academy of Pediatric Dentistry (AAPD) published guidelines for BGTs; however, there are currently no specific recommendations or BGT guidelines for managing autistic patients during dental treatment (AAPD 2020).

Dental treatment for children involves three-way communication between the dentist, patient, and parent(s), and not all BGTs can be applied to autistic patients or will satisfy their parents (Dean et al. 2016; AAPD 2020). There are currently few studies on the parental acceptance of BGTs for patients with special healthcare needs (SHCN), including autism. Studies from 1995 to 2013 reported that general anaesthesia (GA) was the least acceptable BGT, while positive reinforcement (PR) and distraction (DIS) were nearly universally accepted by parents of SHCN patients (Brandes et al. 1995; Elango et al. 2012; de Castro et al. 2013). However, many recent studies on healthy children have shown that parental acceptance of BGTs continues to evolve, with increasing acceptance of oral sedation (OS) and GA (Peretz et al. 2013; Jafarzadeh et al. 2014; Patel et al. 2016; Al Zoubi et al. 2019). Only one study, conducted by Marshall in USA in 2008, specifically examined parental acceptance of BGTs in autistic patients. The results showed a 100% acceptance rate for PR and tell–show–do (TSD), and over 90% acceptance for DIS, GA, and active restraint by parent (ARBP). Active restraint by staff (ARBS) and passive restraint by device (PRBD) had lower acceptance rates compared to other BGTs (Marshall et al. 2008).

In recent years, parents' views regarding their children have evolved and society have changed due to the influence of the digital era (Jafarzadeh et al. 2014; Patel et al. 2016; Al Zoubi et al. 2019, 2021). Moreover, no study has specifically examined parental acceptance of BGTs for patients with autism, particularly in Asian countries. This study aimed to assess parental acceptance of nine BGTs during dental treatment of their autistic patients in Thailand and to assess the factors affecting parental acceptance of these techniques.

## Materials and methods

This cross-sectional study was approved by the Ethical Committee of the Faculty of Dentistry and Faculty of Pharmacy, Mahidol University, Institutional Review Board (ref. no. COA.No.MU-DT/PY-IRB 2021/022.1702), and was registered with the Thai Clinical Trials Registry (TCTR20220521001).

### Sample of the study

The sample size was determined based on the results of a study conducted by Brandes in the USA in 1995 (Brandes et al. 1995), using the highest standard deviation (SD) of VAS scores for parental acceptance of BGTs in children with special needs to calculate the largest required sample size. Assuming an infinite population mean, the following statistical parameters were applied: SD (*σ*) = 43.40, error (*d*) = 8.30, significance level (*α*) = 0.05, and *Z*(0.975) = 1.96. Based on these assumptions, the minimum required sample size was calculated.

$$n = \frac{{Z_{{1 - \frac{\alpha }{2}}}^{2} \left( \sigma \right)^{2} }}{{d^{2} }}$$

$$n\hspace{0.17em}=\hspace{0.17em}\frac{{{(1.96)}_{1 - \frac{0.05}{2}}^{2}(43.4)}^{2}}{{10}^{2}}$$

$${\mathrm{Sample}}\;{\mathrm{size}}\left( n \right) = 106$$

The estimated sample size was 106 participants in total. Data for this study were collected via an online questionnaire due to the COVID-19 pandemic in Thailand.

Parents of autistic individuals aged < 20 years who were patients at the Mahidol University Dental Hospital and members of autism online community in Thailand were recruited for the study. Parents who were blind, deaf, had language impairments, or lacked the ability to read or understand Thai were excluded from the study. The parents were contacted by phone, and the study procedures including objectives were explained by a single operator. Informed consent was sent to all parents prior to their enrolment in the study, and they were given the opportunity to ask any questions.

The BGTs used in this study included both basic and advanced BGTs, following the 2020 AAPD guidelines (AAPD 2020). Advanced BGTs focused on protective stabilization and pharmacological management. Protective stabilization can be performed by staff member or a parent, with or without the use of a stabilization device. In this study, protective stabilization was subdivided into the categories of active restraint by parent (ARBP), active restraint by staff (ARBS), and passive restraint by device (PRBD).

The participating parents completed a questionnaire administered via SurveyMonkey.com (SurveyMonkey Inc., California, USA), which comprised two sections. The first section collected demographic information related to both the parents and their autistic children, including the parent's age, gender, educational attainment, and monthly income. For the autistic child, data were gathered on age, gender, autistic level, history of dental visits (yes or no), and prior experience with BGTs, including which techniques had been previously utilized. The second section assessed parental acceptance of nine BGTs currently employed in dental management: tell–show–do (TSD), positive reinforcement (PR), distraction (DIS), nitrous oxide/oxygen inhalation (NOOI), active restraint by parent (ARBP), active restraint by staff (ARBS), passive restraint by device (PRBD), oral sedation (OS), and general anaesthesia (GA). All participating parents rated their acceptance of each BGT using a visual analog scale (VAS) after watching video clips. The BGT videos were created by paediatric dentistry residents. In each video, one resident portrayed the dentist applying a specific behaviour guidance technique, while another acted as a simulated patient in a dental setting, displaying potentially cooperative behaviour to represent autistic patients. Each clip was under 30 s and demonstrated the application of a single BGT. Thai narration was included to explain the procedure, and the narration script was approved by the Ethical Committee of the Faculty of Dentistry and Faculty of Pharmacy, Mahidol University, Institutional Review Board. Prior to use, the videos were reviewed by three board-certified paediatric dentists to ensure accuracy and appropriateness for research purposes. The BGT clips were randomly organized into six sets using a computer program, with only brief descriptions provided for each technique.

The VAS ranged from 0 to 100, indicating levels from "Totally Disagree" to "Totally Agree" about the use of BGTs with their autistic child. After watching each BGT, parents rated their level of acceptance using the VAS and continued this process until all nine BGTs were completed.

The validity of the questionnaire was evaluated using the Index of Item Objective Congruence (IOC) by three board-certified paediatric dentists. The IOC scores for each question ranged from 0.67 to 1.00. For reliability, 10 parents completed the questionnaire, followed by a retest one month later. The intraclass correlation coefficient values ranged from 0.8 to 1.0.

### Statistical analysis

The Statistical Package for the Social Sciences (SPSS Inc., IBM, Chicago, IL, USA) for Mac version 29.0 was utilized in this study. Mean VAS scores were calculated to rank the acceptance of each BGT. Friedman’s two-way analysis of variance was employed to analyse differences in VAS scores among the BGTs. The Mann–Whitney *U* test was used to compare VAS scores between groups based on the parents’ genders, the genders of autistic patients, past dental experiences of the autistic patients, and their experiences with BGTs. The Kruskal–Wallis test was applied to compare VAS scores across groups based on parents’ ages, education levels, socio-economic status, as well as the ages and the level of autism of the parent’s child. For post hoc comparisons following the Kruskal–Wallis and Friedman tests, pairwise comparisons with Bonferroni correction were employed. Furthermore, linear regression was used to rank the importance of each factor affecting the acceptance level. A confidence level of 95% was established, with a p-value of < 0.05 considered statistically significant.

## Results

A total of 110 parents of autistic patients completed both parts of the online questionnaire. The demographic data of the parents are presented in Table 1, while information about the autistic patients is presented in Table 2.

**Table 1:** Demographic data of parents and income

Demographic (*N* = 110)	Number (%)
Parent’s	
Age	
Generation Z (< 29 years)	1 (0.9)
Generation Y (29–44 years)	37 (33.6)
Generation X (45–60 years)	64 (58.2)
Baby boomer (> 60 years)	8 (7.3)
Gender	
Male	14 (12.7)
Female	96 (87.3)
Education level	
< Bachelor’s	48 (43.6)
Bachelor’s	55 (50.0)
> Bachelor’s	7 (6.4)
Income (per month)	
< 500 USD	63 (57.3)
500 ≤ 800 USD	19 (17.3)
800 ≤ 1300 USD	16 (14.5)
> 1300 USD	12 (10.9)

**Table 2:** Demographic data of autistic persons

Demographic (*N* = 110)		Number (%)	
Autistic person’s			
Age			
Preschool (3–5 years)		14 (12.7)	
School age (6–12 years)		55 (50.0)	
Adolescent (13–18 years)		37 (33.6)	
Adult (19–20 years)		4 (3.6)	
Gender			
Male		87 (79.1)	
Female		23 (20.9)	
Autistic level			
Level 1 “Requiring support”		32 (29.1)	
Level 2 “Requiring substantial support”		44 (40.0)	
Level 3 “Requiring very substantial support”		34 (30.9)	
Past dental experience			
No experience		14 (12.7)	
Has experience		96 (87.3)	
BGTs experience			
ARBP experience			60 (54.5)
PR experience			57 (51.8)
PRBD experience			56 (50.9)
ARBS experience			52 (47.3)
TSD experience			39 (35.5)
DIS experience			33 (30.0)
GA experience			22 (20.0)
OS experience			5 (4.5)
NOOI experience			2 (1.8)

*BGTs* behaviour guidance techniques, *ARBP* active restraint by parent, *PR* positive reinforcement, *PRBD* passive restraint by device, *ARBS* active restraint by staff, *TSD* tell–show–do, *DIS* distraction, *GA* general anaesthesia, *OS* oral sedation, *NOOI* nitrous oxide/oxygen inhalation

The majority of parents were generation *X* (58.2%) and female (87.3%). Most had a bachelor’s degree (50.0%) or a lower level of education (43.6%), and the majority reported a monthly income below $500 USD (57.3%). Half of the parents’ autistic patients were of school age (6–12 years, 50.0%), and most were male (79.1%). Autism severity was classified using a standardized questionnaire, with 40.0% of participants requiring substantial support (Level 2) and 30.9% requiring very substantial support (Level 3).

Most autistic patients had prior dental treatment experience (87.3%) and their experiences with specific BGTs varied. ARBP was the most commonly experienced BGT (54.5%), followed by PR (51.8%) and PRBD (50.9%), while OS (4.5%) and NOOI (1.8%) were the least experienced.

### Parental acceptance of nine BGTs for autistic patients in dental practice

The mean VAS scores were ranked from highest to lowest, with all BGTs receiving scores above 60 (ranging from 60.55 to 94.65), as shown in Table 3. The ranking of the most accepted to the least accepted BGTs was as follows: PR, DIS, TSD, PRBD, ARBS, ARBP, GA, OS, and NOOI. PR had a significantly higher acceptance than other BGTs and all pharmacological BGTs (GA, OS, and NOOI) had a significantly lower acceptance than PR, DIS, TSD, and PRBD, but the acceptance rates were not significantly different when compared to acceptance of active restraint techniques (ARBP and ARBS).

**Table 3:** Mean, standard deviation (SD), median, and interquartile range (IQR) of VAS rated for parental acceptance of each BGTs

BGTs	Means ± SD	Median (IQR)	Range
PR	94.65 ± 12.5	100.00 (3)^a^	20–100
DIS	84.89 ± 21.3	99.50 (29)^b^	0–100
TSD	83.89 ± 21.5	92.5 (27)^b^	0–100
PRBD	76.08 ± 30.9	90.0 (37)^b,c^	0–100
ARBS	71.64 ± 30.9	80.0 (46)^c,d^	0–100
ARBP	67.90 ± 34.1	80.0 (53)^d^	0–100
GA	66.48 ± 33.7	79.0 (57)^d,e^	0–100
OS	66.03 ± 29.6	66.5 (50)^d,e^	0–100
NOOI	60.55 ± 32.5	64.0 (50)^d,e^	0–100

^a^^−^^e^Different letters indicate significant difference among BGTs

*BGTs* behaviour guidance techniques, *PR* positive reinforcement, *DIS* distraction, *TSD* tell–show–do, *PRBD* passive restraint by device, *ARBS* active restraint by staff, *ARBP* active restraint by parent, *GA* general anaesthesia, *OS* oral sedation, *NOOI* nitrous oxide/oxygen inhalation

### Association of parental and socio-environmental factors with VAS

There was no association found between the VAS scores for the nine BGTs and the factors of parental age, gender, or monthly income. However, parents with an education level below a bachelor’s degree showed a significantly higher acceptance of DIS, NOOI, ARBS, PRBD, and GA compared to those with a bachelor’s degree. Additionally, there were no significant differences in the VAS scores among those with a degree higher than a bachelor’s and those with either lower than a bachelor’s or those with a bachelor’s degree, as shown in Table 4.

**Table 4:** Median and interquartile range (IQR) of VAS for parental acceptance of each BGT by education level of parents

BGTs	Education level [Median (IQR)]		
	< bachelor’s (*n* = 48)	Bachelor’s (*n* = 55)	> bachelor’s (*n* = 7)
TSD	100.0 (25)	91.0 (28)	80.0 (15)
PR	100.0 (0)	100.0 (5)	100.0 (20)
DIS	100.0 (17)^a^	90.0 (30)^b^	84.0 (40)^a,b^
NOOI	78.5 (20)^a^	51.0 (54)^b^	55.0 (20)^a,b^
ARBP	80.5 (50)	74.0 (65)	82.0 (65)
ARBS	90.0 (28)^a^	70.0 (60)^b^	78.0 (37)^a,b^
PRBD	96.5 (22)^a^	80.0 (60)^b^	83.0 (20)^a,b^
OS	80.0 (50)	60.0 (39)	72.0 (35)
GA	90.0 (55)^a^	60.0 (56)^b^	70.0 (18)^a,b^

^a^^−^^b^Different letters indicate significant difference among education level

*BGTs* behaviour guidance techniques, *TSD* tell–show–do, *PR* positive reinforcement, *DIS* distraction, *NOOI* nitrous oxide/oxygen inhalation, *ARBP* active restraint by parent, *ARBS* active restraint by staff, *PRBD* passive restraint by device, *OS* oral sedation, *GA* general anaesthesia

### Association of factors related to autistic patients and VAS

There was no association found between the VAS scores for the nine BGTs and the age or gender of the autistic patients. Parents of autistic patients with no prior dental treatment experience showed a significantly higher acceptance rate for OS and GA compared to those whose children had previous dental experiences, as shown in Table 5.

**Table 5:** Median and interquartile range (IQR) of VAS for parental acceptance of each BGT by past dental experience

BGTs	Past dental experience [Median (IQR)]		
	No exp (*n* = 14)	Exp (*n* = 96)	*p* value
TSD	97.0 (21)	91.0 (30)	0.508
PR	100.0 (8)	100.0 (1)	0.556
DIS	95.0 (22)	99.5 (30)	0.870
NOOI	85.0 (40)	60.0 (47)	0.083
ARBP	90.5 (25)	75.5 (61)	0.076
ARBS	87.0 (50)	80.0 (47)	0.597
PRBD	84.5 (33)	90.0 (46)	0.659
OS	100.0 (30)	63.5 (43)	0.006*
GA	100.0 (23)	74.5 (61)	0.008*

*BGTs* behaviour guidance techniques, *exp* past dental experience, *TSD* tell–show–do, *PR* positive reinforcement, *DIS* distraction, *NOOI* nitrous oxide/oxygen inhalation, *ARBP* active restraint by parent, *ARBS* active restraint by staff, *PRBD* passive restraint by device, *OS* oral sedation, *GA* general anaesthesia

**Fig. 1:**
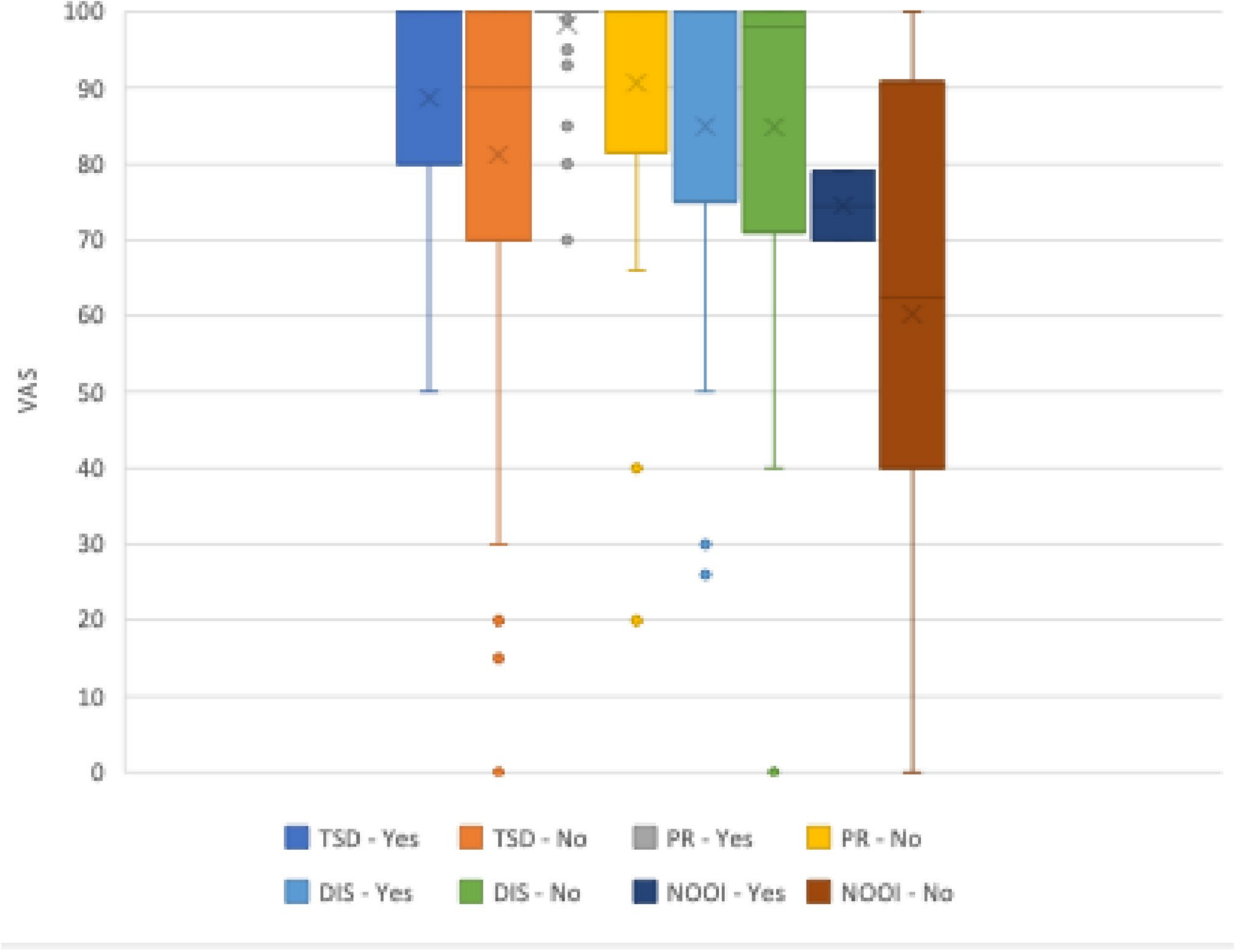
Median of VAS for parental acceptance of basic BGTs by their experience. The * indicates significant difference among groups. *BGTs* behaviour guidance techniques, *TSD* tell–show–do, *PR* positive reinforcement, *DIS* distraction, *NOOI* nitrous oxide/oxygen inhalation, *Yes* had experience, *No* no experience

**Fig. 2:**
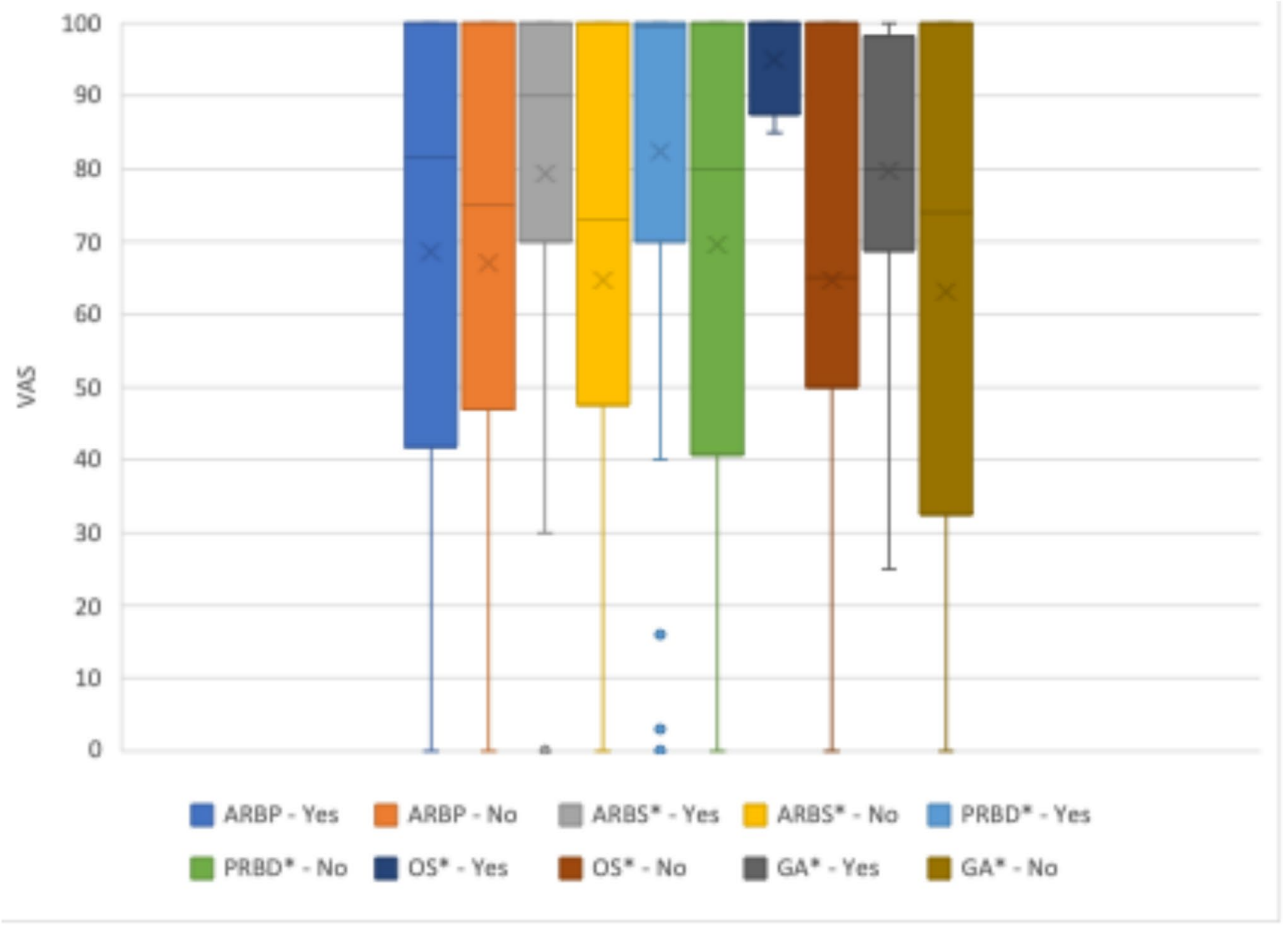
Median of VAS for parental acceptance of advanced BGTs by their experience. The * indicates significant difference among groups. *BGTs* behaviour guidance techniques, *ARBP* active restraint by parent, *ARBS* active restraint by staff, *PRBD* passive restraint by device, *OS* oral sedation, *GA* general anaesthesia, *Yes* had experience, *No* no experience

Regarding past experiences with BGTs, there was no association between the VAS scores and basic BGT experience, as illustrated in Fig. 1. In contrast, experience with advanced BGTs showed a different trend. Parents of autistic patients who had previously received advanced BGTs (except for ARBP) rated their acceptance levels as significantly higher for those techniques as compared with parents with no such experience, as depicted in Fig. 2.

Additionally, parents of patients with autism Level 3 rated their acceptance of PRBD and ARBS as significantly higher than parents of patients with autism Level 2, as presented in Table 6. Moreover, parents of patients with autism Level 3, who require substantial support in daily life, judged PR as being unsuitable for their children, which was significantly different from the perceptions of parents of patients with autism Level 1.

**Table 6:** Median and interquartile range (IQR) VAS for parental acceptance of each BGT by autistic level

BGTs	Autism level [Median (IQR)]		
	Level 1 (*n* = 32)	Level 2 (*n* = 44)	Level 3 (*n* = 34)
TSD	100.0 (14)^a^	90.0 (26)^a,b^	87.0 (46)^b^
PR	100.0 (3)	100.0 (1)	100.0 (15)
DIS	100.0 (27)	95.5 (29)	99.5 (29)
NOOI	60.0 (38)	65.0 (69)	70.0 (50)
ARBP	81.5 (57)	75.5 (65)	85.0 (50)
ARBS	79.0 (46)^a,b^	72.5 (57)^a^	90.0 (32)^b^
PRBD	87.5 (50)^a,b^	80.0 (49)^a^	100.0 (20)^b^
OS	60.5 (41)	69.0 (45)	70.0 (61)
GA	70.0 (49)	80.0 (55)	82.0 (63)

^a^^−^^b^Different letters indicate significant difference among autism level

*BGTs* behaviour guidance techniques, *TSD* tell–show–do, *PR* positive reinforcement, *DIS* distraction, *NOOI* nitrous oxide/oxygen inhalation, *ARBP* active restraint by parent, *ARBS* active restraint by staff, *PRBD* passive restraint by device, *OS* oral sedation, *GA* general anaesthesia

### Regression analysis of predictors for parental acceptance of BGTs

Linear regression analysis was conducted to determine the impact of various factors on parental acceptance of BGTs for autistic children. The results showed that past BGT experience was the most influential factor in increasing parental acceptance. This refers specifically to the advanced BGT that had previously been used with the child. All advanced BGTs demonstrated increased parental acceptance when there was past experience with that specific technique, except for ARBP.

For BGTs influenced by more than one factor, the significant predictors could be ranked as follows: past BGT experience with the specific BGT, past dental experience, autism level, and parent’s educational level. Parental acceptance scores were higher when the specific advanced BGT had been previously used with the child. In contrast, children with prior dental treatment experience were associated with lower parental acceptance scores, and parents with higher educational levels also tended to report lower acceptance levels for BGTs.

## Discussion

Basic BGTs were generally well accepted, with the exception of NOOI, which received the lowest acceptance rate. Advanced BGTs, including ARBS, PRBD, OS, and GA, were highly accepted, particularly among parents of autistic patients with prior experience using these techniques. However, parental ratings exhibited a wide range of acceptance, spanning from 0 to 100, except for PR, which had a narrower range of 20 to 100. Overall, this study found that the acceptance of BGTs among the participating Thai parents of autistic patients varied but generally leaned towards overall acceptance.

In this study, the VAS scores for basic BGTs (with the exception of NOOI) were more favourable than those for advanced BGTs. This finding aligns with previous studies conducted on healthy children (Jafarzadeh et al. 2014; Seangpadsa et al. 2020) and autistic patients (Marshall et al. 2008). However, NOOI was noted as having the lowest acceptance rate, which contrasts with Marshall's study that reported higher acceptance of NOOI compared to ARBS and PRBD among parents of autistic children (Marshall et al. 2008) as well as a study in healthy children (Al Zoubi et al. 2021). One possible explanation for the lower acceptance of NOOI in our study is that only 1.8% of parents of autistic patients had prior exposure to this technique, resulting in a lack of familiarity and, consequently, limited trust in it. In Thailand, dental settings generally do not offer NOOI; only a limited number of dental hospitals or private clinics provide this option. Furthermore, contemporary pharmacological BGTs are recognized for their high safety and reliability, and an increasing number of autistic patients have had prior positive experiences with these techniques. As a result, OS and GA have been gaining greater acceptance over time. (Jarfazadeh; Eaton et al. 2005).

The factors influencing the parental acceptance of BGTs included both parental and autistic patients’ demographic characteristics. Interestingly, socio-economic status did not appear to impact the selection of BGTs, which contrasts with findings from previous studies (Lawrence et al. 1991; Havelka et al. 1992; Boka et al. 2014; Patel et al. 2016). In Thailand, all autistic patients have access to similar medical welfare programmes for patients with SHCN; therefore, socio-economic factors may not significantly affect parental acceptance of BGTs in this context.

Moreover, the study found that parents with an education level lower than a bachelor’s degree demonstrated greater acceptance of DIS, NOOI, ARBS, PRBD, and GA. Previous research has similarly shown that higher levels of parental education are associated with lower acceptance of PRBD (Boka et al. 2014). Many parents also tend to prefer ARBP over ARBS, possibly due to concerns that restraint by dental staff may cause physical or emotional harm to their child. This trend may reflect differing perceptions of the appropriateness and necessity of various BGTs based on educational background. Parents with lower levels of formal education might prioritize immediate and practical solutions for managing their child’s dental treatment, whereas those with higher education levels may place greater emphasis on long-term psychological impacts. Overall, these findings underscore the complexity of factors influencing parental acceptance of BGTs and highlight the importance of tailoring communication and treatment planning to account for educational and socio-economic differences among families.

Furthermore, parents of patients with autism Level 3 expressed higher acceptance of ARBS and PRBD. It was possibly due to the uncontrolled behaviours associated with this level of autism, which necessitate physical restraint to prevent accidents during dental treatment. This corresponds with the general tendency for parents to be more accepting of advanced BGTs when managing uncooperative behaviour in children (Peretz et al. 2013; Al Zoubi et al. 2021). Conversely, parents of patients with autism Level 1 demonstrated significantly higher acceptance of the TSD technique as compared with parents of patients with autism Level 3. This difference may stem from an understanding that patients with autism Level 3 often struggle with communication and face barriers that make TSD less effective for them.

Prior experience with BGTs significantly increased acceptance rates for advanced techniques, such as ARBS, PRBD, OS, and GA, which was consistent with findings from previous studies involving both patients with SHCN and healthy children (elBadrawy and Riekman 1986; Frankel 1991; Marshall et al. 2008; Patel et al. 2016; Seangpadsa et al. 2020). Advanced BGTs are often perceived as more invasive, making them more challenging to accept on the first attempt. However, following a successful treatment experience with these techniques, acceptance levels tend to increase.

Interestingly, ARBP, a technique that many parents may have previously employed with their autistic child, had a lower acceptance rate as compared with other advanced BGTs. Having experience with ARBP did not significantly influence parents’ acceptance of this technique. Autistic patients often require parental restraint to manage daily activities such as hair washing, showering, tooth brushing, hair cutting, and even receiving injections. However, some parents may not recognize that ARBP is a familiar BGT that their child has encountered before. This suggests that parents may prefer other advanced BGTs over ARBP, despite their child’s previous experiences with it.

Only the parental acceptance of pharmacological BGTs, specifically OS and GA, showed significant differences based on prior dental experiences. Parents and their autistic children who had previously undergone both nonpharmacological and pharmacological management tended to endure dental procedures without the need for any medical prescriptions. To assess the impact of prior dental experiences, parents of autistic patients were recruited from Mahidol University Dental Hospital and from an autism Facebook group.

The findings suggest that parental acceptance of BGTs, influenced by multiple factors, can be interpreted in light of the relative strength of each predictor. Among these, prior experience with the specific BGT emerged as the most influential factor, indicating that familiarity with the technique may enhance parental trust and acceptance. This is consistent with previous research showing that parents are more likely to approve of techniques they have previously encountered (elBadrawy and Riekman 1986; Frankel 1991; Marshall et al. 2008; Patel et al. 2016; Seangpadsa et al. 2020).

In contrast, a history of prior dental treatment in the child was associated with lower parental acceptance of OS and GA significantly. The supporting reasons are possibly due to previous negative experiences or increased awareness of potential treatment challenges when compared to parent of autistic individuals who had no dental treatment experience. The child’s level of autism also played a role in acceptance, with parents of children requiring higher levels of support more likely to accept certain BGTs, particularly those involving physical restraint. Finally, parents with higher educational attainment tended to report lower acceptance, which may reflect more critical evaluation of the techniques or heightened concern about their appropriateness (Boka et al. 2014).

Limitations of this study include the use of an online questionnaire that did not inquire about specific types of dental treatment, such as urgency, pain, or oral health status/problems, which may have influenced parental acceptance. Several studies have reported that the type of dental treatment, as well as factors like urgency and pain, can affect parental acceptance of BGTs (Patel et al. 2016; Seangpadsa et al. 2020; Al Zoubi et al. 2021). Additionally, the study relied solely on online questionnaires and video clips with brief explanations, which may have affected parents' understanding of each BGT, and there was no protocol in place to control for this variable. Furthermore, the small number of autistic patients with prior experience using NOOI and OS may have affected the reliability of the findings. Since this was a cross-sectional study evaluating parental acceptance of behaviour guidance techniques in Thai autistic patients, the findings reflect only the studied population of Thai parents of autistic patients. Future studies with larger sample sizes and more detailed categorization of dental procedures are recommended to address these limitations and provide more robust conclusions.

## Conclusion

The most widely accepted BGT was PR, while NOOI received the lowest acceptance among the studied population of Thai parents of autistic patients. Factors such as prior BGT experience, past dental experiences, the child’s level of autism, and the parent’s education level should be carefully considered when selecting BGTs for autistic patients.

## Data Availability

No datasets were generated or analysed during the current study.
